# Cavernous Hemangioma of the Uterine Cervix Revealed by Induction Failure: A Rare Case Report

**DOI:** 10.1155/crog/1758571

**Published:** 2026-06-15

**Authors:** Aurélie Buffetau, Hugo Madar, Claire Rozes, Jean Luc Brun, Loïc Sentilhes

**Affiliations:** ^1^ Department of Gynecologic Surgery and Reproductive Medicine, Pellegrin Hospital Group-Aliénor d′Aquitaine Center, Bordeaux University Hospital, Bordeaux, France, chu-bordeaux.fr; ^2^ Department of Obstetrics and Gynecology, Bordeaux University Hospital, Bordeaux, France, chu-bordeaux.fr; ^3^ Service de radiologie et imagerie anténatale de l′enfant et de la femme, Bordeaux University Hospital, Bordeaux, France, chu-bordeaux.fr

## Abstract

Hemangiomas of the uterine cervix are rare benign vascular tumors, with fewer than a hundred cases described in the literature. Their clinical presentation is variable, most often involving abnormal genital bleeding, but they may also be discovered incidentally. Association with pregnancy is reported in a subset of cases, likely reflecting hormonal and hemodynamic influences.

**Case Presentation:**

We report the case of a 29‐year‐old woman with no prior medical history, admitted for induction of labor following intrauterine fetal death at 21 weeks of gestation. Despite multiple attempts with both pharmacologic and mechanical methods of induction, adequate cervical dilation could not be achieved—even under general anesthesia—because the cervix was unusually firm and friable, ultimately necessitating cesarean delivery. Postpartum pelvic MRI and colposcopy revealed a suspicious cervical lesion. Conization with endocervical curettage was performed, and histopathological examination confirmed the diagnosis of cavernous hemangioma of the cervix.

**Conclusion:**

Cervical cavernous hemangiomas are rare and often overlooked in the differential diagnosis of cervical lesions. This case is remarkable because the lesion was revealed by repeated failure of labor induction. Awareness of such unusual presentations may help gynecologists consider vascular tumors in selected obstetric or gynecologic contexts and guide conservative, fertility‐preserving management.

## 1. Introduction

Hemangiomas are benign vascular tumors most commonly found in the skin and liver [1]. Their occurrence in the female genital tract is rare, and involvement of the uterine cervix is exceptionally uncommon. Most cervical hemangiomas are small, asymptomatic, and discovered incidentally [2, 3]. Only a few cases have been reported, making them a diagnostic and therapeutic challenge for gynecologists.

Clinically, cervical hemangiomas may present with abnormal vaginal bleeding or postcoital bleeding or be identified during routine gynecological examination [4, 5].

Because of their rarity and nonspecific presentation, cervical hemangiomas are often overlooked in the differential diagnosis of cervical lesions. Reporting such cases is essential to improve recognition and guide management. We present here a rare case of cavernous hemangioma of the uterine cervix, diagnosed in the postpartum period.

## 2. Case Presentation

The patient was a 29‐year‐old woman from Guinea‐Bissau with no medical history and no prior gynecological follow‐up. She was admitted to the gynecological emergency unit after the incidental discovery of an intrauterine fetal death (IUFD) at 21 weeks of gestation, which was also the occasion of the first recognition of her pregnancy. Ultrasound confirmed the IUFD, showing anhydramnios, with fetal biometrics corresponding to 21 weeks.

Management was initiated with the standard protocol, including oral mifepristone followed 48 h later by induction of labor under epidural analgesia with misoprostol and insertion of cervical dilators (Dilapan). Initial placement of two intracervical dilators, combined with three doses of misoprostol 400 *μ*g every 3 h, failed to achieve cervical ripening and artificial rupture of membranes. A second attempt with four dilators was also unsuccessful, despite the administration of an additional course of misoprostol. Artificial rupture of membranes under ultrasound guidance was attempted without success.

Given the failure of standard induction methods, a second‐line protocol was initiated with intravenous sulprostone (Nalador 500 *μ*g over 8 h). A further attempt at artificial rupture of membranes under ultrasound guidance was again unsuccessful. Therefore, mechanical cervical dilation under general anesthesia was performed using dilators, which allowed successful artificial rupture of membranes and placement of a transcervical balloon. The entire procedure was carried out under continuous ultrasound guidance. Labor induction was then continued with intravenous oxytocin.

Despite 4 days of induction, including multiple pharmacological and mechanical interventions, the cervix remained firm and long, with an external os that was unusually friable and only slightly dilated. The mechanical procedures were technically difficult, with an inability to achieve meaningful cervical dilation and increasing cervical friability with each manipulation, raising concern for tissue fragility. Given the concern for further cervical trauma and potential false passage formation, a multidisciplinary team decision was made to proceed with cesarean section for induction failure in the context of IUFD at 21 weeks in a primigravida. A transverse corporal hysterotomy was carried out, and a stillborn fetus was delivered. The surgery was uneventful, and the postoperative course was simple. No uterine malformation was observed, and the cervical canal was patent from the uterine side. Estimated intraoperative blood loss during cesarean section was 210 mL, with no hemorrhagic complications intraoperatively or postpartum. Cervical bleeding during induction attempts was minimal and not quantified. Given the unusual failure of induction, a pelvic magnetic resonance imaging (MRI) was performed to investigate a possible underlying cause (Figure 1).

**Figure 1:**
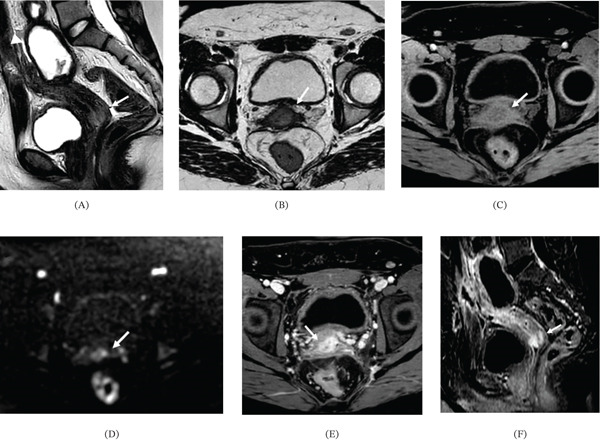
(A) Sagittal and (B) axial T2‐weighted imaging (T2WI) showing a cervical lesion appearing slightly hyperintense (white arrows). Note the periuterine hematoma in the postcesarean section context, appearing hyperintense (arrowhead). (C) Axial T1‐weighted image (T1WI) showing relative hypointensity of the lesion. (D) Diffusion‐weighted imaging (DWI) demonstrating mild hyperintensity of the lesion. (E) Axial and (F) sagittal postcontrast images demonstrating intense delayed enhancement greater than that of the normal cervix.

MRI revealed a low intracervical mass involving the endocervical canal and exocervical surface. The lesion demonstrated slight hyperintensity on T2‐weighted images, relative hypointensity on T1‐weighted images, and mild diffusion hyperintensity. It remained strictly confined to the cervix, without invasion of the vaginal walls or parametrium, with continuity of the peripheral T2‐hypointense ring corresponding to the cervical fibrous stroma. The cavity line was clearly visible. Following contrast administration, the lesion exhibited intense delayed enhancement greater than that of the adjacent cervix. These imaging features raised the suspicion of a localized cervical neoplasm (FIGO Stage IB2).

Colposcopy showed a friable, exophytic cervix, and biopsies, along with a Pap smear, were performed. Cytology was normal, and histology revealed ulcerated exocervical mucosa with fleshy granulation tissue but no condylomatous, dysplastic, or malignant features.

Due to the discrepancy between clinical and imaging findings suggestive of cervical neoplasia and the normal pathology results, the case was reviewed at a multidisciplinary tumor board, which recommended diagnostic conization to rule out endocervical cancer. Conization combined with endocervical curettage was performed under general anesthesia. Perioperative hemostasis was achieved using monopolar ball coagulation. Blood loss was minimal, and the postoperative course was uncomplicated. Although vascular lesions may carry a bleeding risk, no hemorrhagic complication occurred in this case. Definitive histopathological analysis revealed a vascular proliferation consistent with cavernous hemangioma. The (Figure 2) endocervical curettage was unremarkable.

**Figure 2:**
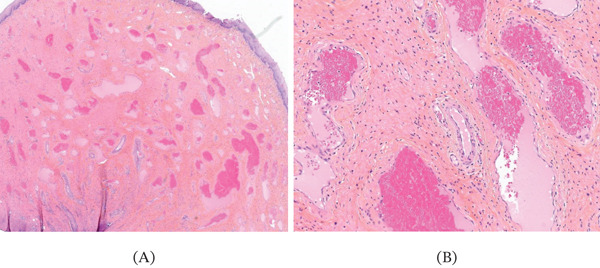
Histopathological examination of the cervical lesion. (A) Low‐power view (H&E) showing multiple dilated vascular spaces within the cervical stroma. (B) High‐power view (H&E) demonstrating blood‐filled vascular channels lined by bland endothelial cells without atypia or mitotic activity.

## 3. Discussion

Cavernous hemangioma of the cervix is an exceptionally rare benign vascular tumor [1]. Only a few cases have been reported, and approximately 23 were described in association with pregnancy [6]. Histologically, cervical hemangiomas are characterized by dilated vascular channels lined by endothelial proliferation, and they are classified as either capillary or cavernous types [7, 8]. Their pathophysiology is not completely understood, although hormonal influence, particularly estrogen and progesterone, appears to play a role in their development and growth [7]. Several reports have described lesion enlargement during pregnancy and regression in the postpartum period, supporting a hormonal and hemodynamic contribution [6, 7, 9, 10].

Clinical manifestations vary, ranging from incidental findings to abnormal vaginal bleeding, postcoital bleeding, or obstetric complications (abnormal uterine bleeding, postcoital bleeding, postpartum hemorrhage, and disseminated intravascular coagulation) [6, 8]. In one review, more than half of symptomatic patients presented with abnormal uterine bleeding, while a smaller proportion reported postcoital bleeding. A significant proportion of cases, however, remain asymptomatic and are discovered only during histological examination [4, 5].

Our patient presented in a particularly unusual way, as the lesion was revealed in the context of failed induction of labor for IUFD at 21 weeks. Despite multiple attempts using pharmacologic and mechanical methods, cervical dilation could not be achieved, raising suspicion of an underlying structural abnormality. Subsequent MRI and colposcopy suggested a cervical lesion, and histopathological examination after conization confirmed the diagnosis of cavernous hemangioma. To our knowledge, very few cases of cervical hemangioma have been reported with induction failure as the initial presentation, making this case noteworthy.

Several reported cases of uterine and cervical hemangiomas in the literature have attempted to describe their MRI features. Uterine hemangioma is typically characterized as an intramyometrial mass with T2 hyperintensity, slight T1 hypointensity, and progressive centripetal enhancement leading to homogenization. The signal may appear heterogeneous due to hemorrhagic changes and calcifications (commonly seen in venous malformations related to thrombosis).

The presence of multiple linear low‐signal strands on T2‐weighted or postcontrast T1‐weighted images has been described as a characteristic feature [11], although, in our case, these were barely visible, likely due to the small lesion size.

Another feature observed, herein, which could help differentiate hemangioma from carcinoma and has not been previously described, is the presence of delayed postcontrast enhancement greater than that of the adjacent cervix. In contrast, in untreated cervical neoplasia, the cervical mass typically enhances less than the normal myometrium.

Management of cervical hemangiomas depends on clinical presentation, patient age, and fertility considerations. Conservative approaches are preferred, including local excision, conization, cauterization, or laser photocoagulation [6, 8, 12]. Hysterectomy has historically been performed in some cases, particularly in women without reproductive desire, but it is now generally reserved for refractory or complicated presentations [13]. Ideally, diagnostic conization should be avoided in asymptomatic patients, with diagnosis established through colposcopy and imaging whenever possible. Importantly, no malignant transformation or recurrence has been described in the literature, and the prognosis remains excellent.

## 4. Conclusion

Cavernous hemangioma of the cervix is a rare benign vascular lesion that may pose diagnostic and therapeutic challenges, particularly in obstetric settings. This case is unusual in that the hemangioma was revealed by repeated induction failure in the setting of IUFD. Awareness of this rare entity is important, as early suspicion and appropriate investigation can prevent unnecessary delays and guide optimal management. Reporting such cases contributes to improved recognition of cervical hemangiomas and reinforces the value of conservative, fertility‐preserving treatment strategies whenever possible.

## Funding

No funding was received for this manuscript.

## Consent

All the patients allowed personal data processing, and informed consent was obtained from all individual participants included in the study.

## Conflicts of Interest

The authors declare no conflicts of interest.

## Data Availability

The data that support the findings of this study are available from the corresponding author upon reasonable request.
